# Phase Separation Drives SARS-CoV-2 Replication: A Hypothesis

**DOI:** 10.3389/fmolb.2022.893067

**Published:** 2022-05-11

**Authors:** Andrea Vandelli, Giovanni Vocino, Gian Gaetano Tartaglia

**Affiliations:** ^1^ Department of Biochemistry and Molecular Biology, Universitat Autònoma de Barcelona, Barcelona, Spain; ^2^ Universitat Pompeu Fabra (UPF), Barcelona, Spain; ^3^ Department of Pharmacy and Biotechnology, University of Bologna, Bologna, Italy; ^4^ Center for Human Technologies, Istituto Italiano di Tecnologia, Genova, Italy; ^5^ Department of Biology ‘Charles Darwin’, Sapienza University of Rome, Rome, Italy; ^6^ Institucio Catalana de Recerca i Estudis Avançats (ICREA), Barcelona, Spain

**Keywords:** viral RNA, phase separation, stress granules, protein-RNA interactions, RNA-binding proteins

## Abstract

Identifying human proteins that interact with SARS-CoV-2 genome is important to understand its replication and to identify therapeutic strategies. Recent studies have unveiled protein interactions of SARS-COV-2 in different cell lines and through a number of high-throughput approaches. Here, we carried out a comparative analysis of four experimental and one computational studies to characterize the interactions of SARS-CoV-2 genomic RNA. Although hundreds of interactors have been identified, only twenty-one appear in all the experiments and show a strong propensity to bind. This set of interactors includes stress granule forming proteins, pre-mRNA regulators and elements involved in the replication process. Our calculations indicate that DDX3X and several editases bind the 5′ end of SARS-CoV-2, a regulatory region previously reported to attract a large number of proteins. The small overlap among experimental datasets suggests that SARS-CoV-2 genome establishes stable interactions only with few interactors, while many proteins bind less tightly. In analogy to what has been previously reported for *Xist* non-coding RNA, we propose a mechanism of phase separation through which SARS-CoV-2 progressively sequesters human proteins hijacking the host immune response.

## Introduction

Identification of viral interactions within the host cell can lead to the design of novel strategies against infection. Recently, different high-throughput strategies have been implemented to characterize host interactions with SARS-CoV-2 proteins and genomic RNA.

Non-structural proteins of SARS-CoV-2 have been used for affinity purification to retrieve host binding partners using mass spectrometry in HEK-293T/17 cells (Gordon et al., 2020). A total of 332 interactions between human and SARS-CoV-2 proteins have been identified. Around 40% of SARS-CoV-2 interacting proteins are associated with vesicle trafficking pathways and endomembrane compartments.

Here, we focus on four experimental studies aiming to characterize interactions with SARS-CoV-2 genomic RNA.

In one experiment, a multi-omic approach was employed to identify which viral and human RNA-binding proteins (RBPs) are involved in SARS-CoV-2 infection (Kamel et al., 2021). The “comparative RNA interactome capture” (cRIC) method was developed to find in which way the RNA-bound proteome responds to the infection. The results show that SARS-CoV-2 genome is the epicenter of critical interactions with host proteins: many cellular RBP networks are remodeled upon SARS-CoV-2 infection and around 300 proteins are affected, mostly related to RNA metabolic processes and antiviral defenses. A second approach called “viral RNA interactome capture” (vRIC) was employed to identify cellular and viral proteins interacting with SARS-CoV-2 genomic RNA (Kamel et al., 2021). Inhibition of specific proteins interacting with viral RNA was shown to impair SARS-CoV-2 infection.

In another study (Lee et al., 2021), the repertoire of host proteins associated with SARS-CoV-2 and HCoV-OC43 genomes was identified. The work relies on a robust nucleoprotein (RNP) capture protocol. More than 100 host factors directly binding to SARS-CoV-2 RNA were detected. By applying RNP capture on HCoV-OC43, evolutionary conserved interactions between the viral RNAs and the host proteins could be identified. Upon knockdown experiments and transcriptome analysis, Lee *et al.* identified 17 antiviral and 8 pro-viral RBPs that have a role in several steps of the mRNA life cycle. The authors identified La-related protein 1 (LARP1), a downstream target of the mTOR signaling pathway, as an important antiviral host factor that interacts with SARS-CoV-2 RNA.

Another group exploited an approach in which a comprehensive identification of RBPs followed by mass spectrometry (ChIRP-MS) led to the identification of host proteins that bind SARS-CoV-2 genomic RNA during active infection (Flynn et al., 2021). The results were corroborated with analyses from three RNA viruses and contributed to characterize the specificity of virus-host interactions. Flynn *et al.* also carried out a series of targeted CRISPR screens that highlighted the fact that a big portion of functional RNA-binding proteins act as host’s protectors from virus-induced cell death. Comparative CRISPR screens, performed across seven RNA viruses, reveal both shared and SARS-specific antiviral factors. By combining the RNA-centric approach and the functional CRISPR screens, the authors found a functional connection between SARS-CoV-2 and mitochondria, showing that this organelle is a platform for antiviral activity.

A slightly different experiment led to the identification of more than 100 human proteins that directly and specifically bind to SARS-CoV-2 RNAs in infected cells, performing RNA antisense purification and mass spectrometry. Schmidt et al*.* linked SARS-CoV-2 interactome with changes in proteome abundance induced by viral infection, identifying cellular pathways relevant to SARS-CoV-2 infections. The authors demonstrated by genetic perturbation that both Cellular Nucleic-acid Binding Protein (CNBP) and LARP1, which are two of the most enriched viral RNA binders, have the ability to restrict SARS-CoV-2 replication in infected cells and provide a general map of their direct RNA contact sites. The authors demonstrated a reduced viral replication rate in two human cell lines after a pharmacological inhibition of three other binding partners (PPIA, ATP1A1, ARP2/3 complex).

As experimental studies require time and resources and are affected by intrinsic limitations (for instance mass-spec cannot identify every protein with the same efficiency), computational methods can be exploited to prioritize candidate targets. We previously used the CROSS method (Delli Ponti et al., 2017) to predict secondary structure content of and the *cat*RAPID approach (Bellucci et al., 2011; Agostini et al., 2013b; Cirillo et al., 2017) to compute >100000 human protein interactions with SARS-CoV-2 genomic RNA (Vandelli et al., 2020). The 5′ and 3′ end of SARS-CoV-2 were found to be highly structured, in agreement with subsequent experimental reports (Manfredonia et al., 2020) and show strong propensity to interact with human proteins. Among the identified interactors we identified there are several RNA editases and ATP-dependent RNA helicases that play a role in viral RNA processing and have a high propensity to participate in large macromolecular complexes. A number of proteins are predicted to be sequestered by SARS-CoV-2 genome and their recruitment contributes is thought to modify both the transcriptional and post-transcriptional regulations of host cells.

Here, we analyzed four experimental and one computational studies on human RBPs interactions with SARS-CoV-2 genomic RNA. We exploited the *cat*RAPID algorithm to estimate the ability of proteins to bind SARS-CoV-2 and identified a tight correlation between the number of experiments in which a specific protein is detected experimentally and its predicted binding affinity. Finally, we propose a model in which SARS-CoV-2 RNA promotes the formation of a phase-separated assembly by sequestering specific human proteins.

## Results

### Interactomes Comparison

To retrieve interactions relevant for SARS-CoV-2 infection, we analysed four protein-RNA interactome experiments (Supplementary Material S1).

**FIGURE 1:**
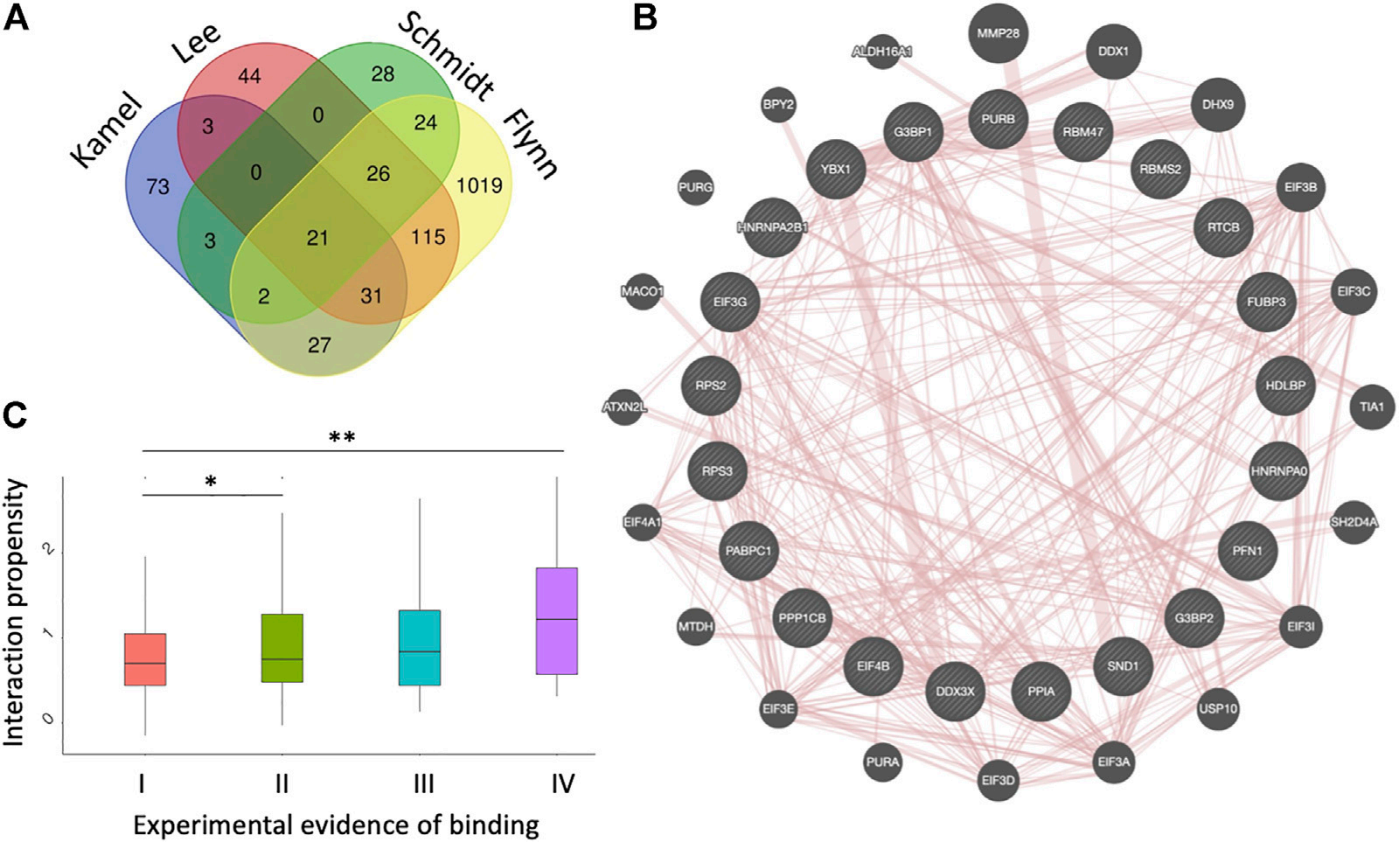
Datasets of protein interactions with SARS-CoV-2 genome. **(A)** experimental datasets (Flynn et al., 2021; Kamel et al., 2021; Lee et al., 2021; Schmidt et al., 2021). The name of each dataset is shown above the diagrams. **(B)** diagram showing the protein-protein interactions among the 21 proteins identified in the four experiments, as annotated by GeneMANIA (Warde-Farley et al., 2010). **(C)** *cat*RAPID interaction scores (Agostini et al., 2013b; Armaos et al., 2021) correlate with the number of experiments reporting a protein to interact with SARS-CoV-2, indicating that strong binding proteins are more likely to be identified; *p-value < 0.05; **p-value < 0.01 (Wilcoxon rank sum test); I II, II, III and IV indicate proteins detected in 1,2,3 or 4 experiments, respectively.

Twenty-one proteins were found in common to the four datasets (Flynn et al., 2021; Kamel et al., 2021; Lee et al., 2021; Schmidt et al., 2021) (Figure 1A). The list includes PABPC1 (Polyadenylate-binding protein 1), SND1 (Staphylococcal nuclease domain-containing protein 1), PPIA (Peptidyl-prolyl cis-trans isomerase A), DDX3X (ATP-dependent RNA helicase DDX3X), HNRNPA2B1 (Heterogeneous nuclear ribonucleoproteins A2/B1), HNRNPA0 (Heterogeneous nuclear ribonucleoprotein A), G3BP1 (Ras GTPase-activating protein-binding protein 1), G3BP2 (Ras GTPase-activating protein-binding protein 2), EIF4B (Eukaryotic translation initiation factor 4B), RPS2 (40S ribosomal protein S2), RPS3 (40S ribosomal protein S3), EIF3G (Eukaryotic translation initiation factor 3 subunit G) and YBX1 (Y-box-binding protein 1), Supplementary Tables S1, S2).

These proteins form a dense protein-protein network (Figure 1B) containing several stress granule components (G3BP1, G3BP2, EIF4B, DDX3X, YBX1, PABPC1), ribosomal units (RPS2 and RPS3) and pre-mRNA processing units (HNRNPA1/B2, HNRNPA0, YBX1) (Warde-Farley et al., 2010). The biological relevance of these interactions is confirmed by the fact that SARS-CoV-2 N protein impairs stress granule by sequestering G3BP1 (Lu et al., 2021; Zheng et al., 2021). RPS2 and RPS3 are important because the NSP1 protein of SARS-CoV-2 is responsible for the impairment of mRNA translation by blocking the entry access to the ribosome. The docking within the ribosomal entry channel occurs through binding with RPS2 and RPS3 together with 18S RNA (Mendez et al., 2021).

Some of these proteins have been shown to be also relevant for other viruses’ infection. SND1 is involved in Epstein-Barr infection (Tong et al., 1995); PABPC1 positively regulates Dengue virus infection (Suzuki et al., 2016); PPIA acts as a mediator for SARS-CoV nucleoprotein during the cell invasion process and stimulates RNA-binding ability of HCV NS5A (Chen et al., 2005; Foster et al., 2011); EIF3G is involved in FCV infection process (Pöyry et al., 2007) and DDX3X has been shown to facilitate the viral replication of other several viruses, such as HIV-1, Dengue, Zyka, Venezuelan equine encephalitis and hepatitis C virus (Yedavalli et al., 2004; Amaya et al., 2016; Doñate-Macián et al., 2018). DDX3X has been identified as a suitable target to fight against SARS-CoV-2 infection by Ciccosanti et al. (2021). More precisely, DDX3X has the capability of unfolding viral RNA secondary structures (Kukhanova et al., 2020) as reported for HIV-1 (Brai et al., 2020) in which it enhances both translation and nucleus-to-cytoplasm transport (Stunnenberg et al., 2018), and West Nile (Brai et al., 2019). DDX3X belongs to the DEAD-box family of ATP-dependent RNA helicases and assumes a crucial role in an important variety of processes concerning RNA metabolism, including transcription, splicing, and initial phase of translation (Ariumi, 2014). Importantly, DDX3X interacts with the N protein of SARS-CoV-2 and is required to infect both Vero E6 and Calu-3 cells (Ciccosanti et al., 2021). Additionally, SARS-CoV-2 protein N interacts with DDX3X to inhibit its activity in the antiviral response (Winnard et al., 2021). For these reasons, treating cells with DDX3X inhibitors represents a promising approach to block SARS-CoV-2 replication and viral production (Maga et al., 2011; Brai et al., 2020).

### Relationship Between Experimental Interactomes and Computational Predictions

We used the *cat*RAPID method to understand the relationship between experimental evidence of binding and predicted interaction propensity that estimates interaction affinity (Agostini et al., 2013a; Cid-Samper et al., 2018). For this analysis we followed a procedure previously introduced to study the interactome of the long non-coding RNA *Xist* (Cirillo et al., 2017). We computed all SARS-CoV-2 interactions with proteins reported in the four experimental datasets and counted how many times they were identified (Supplementary Material S1). We observed a distinct correlation between occurrence and strength of interactions, indicating that high-affinity interactions are more likely to be detected (Figure 1C). We note that in the case of *Xist*, strong interaction proteins were predicted to initiate the formation of a phase-separated assembly (Cerase et al., 2019, 2022), as recently confirmed experimentally (Markaki et al., 2021; Jachowicz et al., 2022).

### Evaluation of the Predictions of SARS-CoV-2 Protein Interactions

The vRIC dataset by Kamel *et al.* contains both enriched and depleted interactions (Kamel et al., 2021) and thus can be used to assess the ability of *cat*RAPID to distinguish between binding and non-binding proteins. To analyze the vRIC interactome, we computed *cat*RAPID predictions of interactions with an experimental FDR <0.10 for SARS-CoV-2 RNA following a procedure detailed in a previous work (Vandelli et al., 2020) (Supplementary Material S1).

**FIGURE 2:**
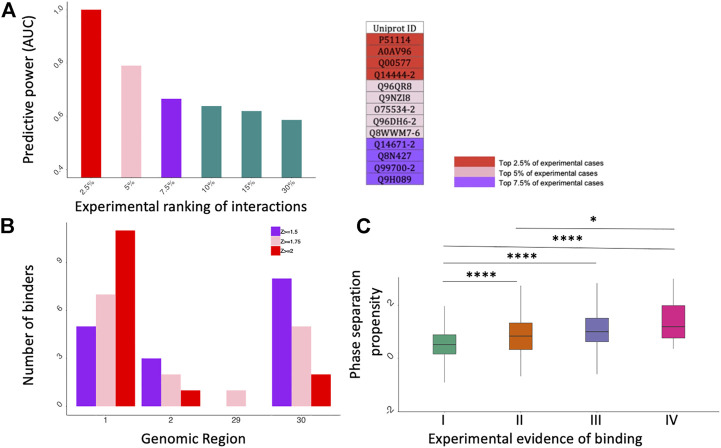
catRAPID and catGRANULE predictions of protein interactions. **(A)** catRAPID performance evaluation. On the *X* axis we report different portions of the experimental dataset ranked by fold change and on the *Y* axis there is the corresponding predictive power (Area Under the ROC Curve, AUC). On the right, we report a summary table showing the Uniprot IDs of top 2.5%, 5% and 7.5% experimental cases. **(B)** Distribution of specific binders for Kamel et al. dataset (Kamel et al., 2021). The most contacted SARS-CoV-2 genomic regions correspond to the 5’ (first fragment) e 3’ (30th fragment). **(C)** catGRANULE phase separation propensity scores correlate with the number of experiments reporting a protein to interact with SARS-CoV-2 (Bolognesi et al., 2016; Cid-Samper et al., 2018); *p-value < 0.05; ****p-value < 0.0001 (Wilcoxon rank sum test); I II, II, III and IV indicate proteins detected in 1,2,3 or 4 experiments, respectively.

As shown in Figure 2A, *cat*RAPID performs extremely well when the proteins are ranked according to their experimental scores (fold change; Supplementary Table S3): the predictive power is proportional to the significance of protein interactions: the Area Under the ROC Curve (AUC) increases from 0.60 to 0.99 while the experimental scores move from 30% (i.e., the 30% strongest positives vs. the 30% strongest negatives) to 2.5% (i.e., the 2.5% strongest positives vs. the 2.5% strongest negatives). Thus, in agreement with the results presented in Figure 1C, computational approaches such as *cat*RAPID can be exploited to address the problem of which proteins bind more tightly to SARS-CoV-2 genome.

### Specific Binders to SARS-CoV-2 Genomic Fragments

*cat*RAPID was employed for the localization of protein binding sites on SARS-CoV-2 genomic RNA. To identify which regions of SARS-CoV-2 bind to specific proteins, we computed interactions for the four experimental protein datasets (30 fragments; Supplementary Material S4), a procedure already proven to be efficient in a previous work (Vandelli et al., 2020).

For each dataset the proteins bound to one fragment at a certain interaction threshold were retained as interactors. We applied three Z-score thresholds (Z ≥ 1.5, Z ≥ 1.75 and Z ≥ 2) in order to evaluate the binding at the different levels of stringency. Higher Z-scores correspond to higher interaction strength (Supplementary Material S5).

Regions encompassing nucleotides 1–1000, 1001–2000, 22001–23000, 26001–27000, 28001–29000, 29001–29903 (Fragments 1, 2, 23, 27, 29 and 30 respectively) are the most contacted SARS-CoV-2 regions, with a high number of interactors in fragments 1, 2 and 30. (Figure 2B; Supplementary Figures S1–S3). In particular, fragment 1, corresponding to the 5′ end of SARS-CoV-2 genome, is the region showing the highest number of specific interactors in all four datasets, as previously discovered (Vandelli et al., 2020). DDX3X is the only common interactor reported in the experimental and computational studies. At a Z ≥ 1.75 we DDX3X is found to bind specifically to fragment 1 of SARS-CoV-2.

### Experimental Interactors Have a High Propensity to Phase-Separate

Stress granules facilitate the establishment of an antiviral state by limiting viral protein accumulation and regulating signaling cascades that affect replication (McCormick and Khaperskyy, 2017). The sequestration of G3BP1, G3BP2, EIF4B, DDX3X, YBX1, PABPC1, among other proteins, is part of a mechanism through which SARS-CoV-2 eludes the host immune response by weakening the formation of stress granules (Lu et al., 2021; Zheng et al., 2021). Biochemically, stress granule proteins form labile protein-protein and protein-RNA interactions (Balcerak et al., 2019; Vandelli et al., 2022), which induces the condensation in liquid-liquid phase separated assemblies (Gotor et al., 2020). We reasoned that the relatively small overlap among experimental datasets (Figure 1A) could be caused by the establishment of weak molecular interactions with SARS-CoV-2 RNA. In agreement with this observation, previous studies have suggested that phase separation could be a mechanism through which SARS-CoV-2 attracts host proteins (Iserman et al., 2020; Vandelli et al., 2020).

Using the *cat*GRANULE algorithm to predict phase separation propensities (Bolognesi et al., 2016; Cid-Samper et al., 2018) we analyzed the interactomes of the four experimental datasets. We discovered that the phase separation propensity correlates with how many times proteins are identified experimentally (Figure 2C). Considering that strong binding propensities are associated with proteins reported in the four experiments (Figure 1C) and the reliability of our approach (Figure 2A), we speculate that a possible mechanism of action for SARS-CoV-2 is to target proteins that attract other partners through phase separation.

## Discussion

This work is a comparative analysis on protein-RNA interactomes reported in experimental and computational studies. We found several proteins shared by the four experiments, including PABPC1, SND1, PPIA, EIF3G and DDX3X, which previous studies have shown to regulate replication of viruses.

DDX3X is found in all the experimental studies and it has been proven fundamental in SARS-CoV-2 biological processes and in the replication process of other viruses (Maga et al., 2011; Ariumi, 2014; Stunnenberg et al., 2018; Brai et al., 2019, 2020; Kukhanova et al., 2020; Ciccosanti et al., 2021; Winnard et al., 2021). *cat*RAPID predictions of human protein interactions with SARS-CoV-2 showed a prevalence of specific binders to the 5′ end of the virus, with DDX3X being one of them. Since *cat*RAPID reproduces experimental data to a remarkable extent, as assessed by directly comparing performances at different cut-offs, we believe that this information on the localization of protein interactions is to be taken into account for future analyses.

Predictive studies always have a margin of error, so further work will be necessary for a complete understanding of the specific binding sites and the role(s) of proteins in the context of infection.

In a recent study (Cirillo et al., 2017), we reported that the long non-coding RNA *Xist* physically interacts with few specific proteins that attract several other proteins (Cerase et al., 2019) forming a phase-separated assembly that silences the X chromosome (Cerase et al., 2022; Jachowicz et al., 2022). The relatively poor overlap of interactors among SARS-CoV-2 studies (only 21 proteins in common out of hundreds identified in total) suggests a mechanism similar to the one identified for *Xist*. The fact that SARS-CoV-2 binding proteins are either stress granules components or have high phase separation propensity supports our hypothesis. Indeed, phase separation is caused by weak protein-protein or protein-RNA interactions (Balcerak et al., 2019; Vandelli et al., 2022), which renders the identification of binding partners particularly difficult at the experimental level (Tartaglia, 2016; Cerase and Tartaglia, 2020) and could hamper their reproducibility. Moreover, the fact that proteins with the highest interaction and phase separation propensities were identified in all experimental studies suggests that they could act as the primary attractors to ignite the formation of an assembly that is capable of using host elements for replication. Further work is needed to study this fundamental aspect of SARS-CoV-2 biology and how it could be exploited to prevent viral infection. For example, molecular chaperones (Tartaglia et al., 2010; Alagar Boopathy et al., 2022) could be important players (Guihur et al., 2020) to be investigated in more detail.

## Data Availability

The original contributions presented in the study are included in the article/Supplementary Material, further inquiries can be directed to the corresponding author.

## Supplementary Material

Supplementary Table S1 Twenty-one proteins shared by the four experimental studies. Uniprot IDs and gene names of the proteins are provided.

Supplementary Table S2 Proteins found in each different experimental study.

Supplementary Table S3 Dataset used for the evaluation of *ca*tRAPID performances. For each protein, its Uniprot ID, experimental fold-change and normalized *cat*RAPID score are reported. The top experimental cases are highlighted in different colors according to the relative portion of the dataset.

Supplementary Table S4 Sequence and genomic location of each of the 30 fragments of SARS-CoV-2.

Supplementary Table S5 List of interactors for each experimental dataset, predicted to bind univocally each of the 30 SARS-CoV-2 fragments.
