# Supplementary material for: Phase Separation Drives SARS-CoV-2 Replication: A Hypothesis
Source: Front Mol Biosci. 2022 May 11;9:893067. doi: 10.3389/fmolb.2022.893067 (PMC9132231; doi:10.3389/fmolb.2022.893067)
Supplement: Supplementary file 3 [file DataSheet1.PDF]

Materials and Methods

Datasets used

Five datasets of human SARS-CoV-2 interactions were used in our analysis, four coming from experimental studies (Flynn et al., 2021; Kamel et al., 2021; Lee et al., 2021; Schmidt et al., 2021) and one from a computational study (Vandelli et al., 2020).

Kamel W. et al. dataset

The authors employed a newly developed technique called vRIC, to identify RBPs that interact directly with SARS-CoV-2 RNA (Kamel et al., 2021). A $\log_2$ fold-change > 0 and a FDR of 0.20 was employed to define the set of 160 proteins here analyzed. The comparison of *cat*RAPID performances was performed on both depleted and enriched interactions with a FDR < 0.10 to balance the sets, obtaining a list of 173 proteins.

Lee S. et al. dataset

The authors detected protein-RNA interactions in Vero cells with RAP-MS protocol, both using a full-length gRNA of SARS-CoV-2 and different sub-genomic RNAs (sgRNAs) (Lee et al., 2021). Since Vero cells are an epithelial cell line from the African Green Monkey, we retained only the proteins having a human orthologue. We collected 240 human proteins with FDR < 10%.

Flynn R. A. et al. dataset

The authors carried out a ChIRP-MS experiment in Huh7 and Vero E6 cells at two different time points (24h and 48h from the infection) (Flynn et al., 2021). To build up our dataset, we collected proteins found in Huh7 or Vero cells in one of the two time points. For the interactors found in Vero cells we retrieved those having a human orthologue. The interactome was obtained collecting 1265 proteins with $\log_2$ fold-change ≥ 1.

Schmidt N. et al. dataset

The authors employed the RAP-MS method to identify protein-RNA interactions in Huh7 cells (Schmidt et al., 2021). The interactome contains 104 enriched proteins with FDR < 0.20.

Vandelli A. et al. dataset

Here we previously computationally identified human-virus protein-RNA interactions, through the use of *cat*RAPID *omics* (Vandelli et al., 2020). The SARS-CoV-2 genomic sequence was divided into 30 fragments of 1000 bp and the human interactome was predicted for each fragment. Using the Z-score of the interacting protein-RNA pair as a measure of interaction strength, we generated a high-confidence interactome dataset by including all the proteins bound to any of the 30 fragments of SARS-CoV-2 with different thresholds of stringency.

SARS-CoV-2 genomic sequence

The reference Wuhan sequence with available annotation (EPI_ISL_402119) was downloaded from Global Initiative on Sharing All Influenza Data in March 2020 (GISAID https://www.gisaid.org/). The entire sequence of the virus has been divided in 30 fragments prior to performing catRAPID predictions. This approach turned out to be effective in a previous publication (Vandelli et al., 2020) to specifically identify preferential binding sites on SARS-CoV-2 genome of human proteins, helping also to avoid limitations of the algorithm in handling the full-length genomic sequence

Protein-RNA interaction predictions

The interactions among the 30 fragments of SARS-CoV-2 sequence and the proteomes from the different datasets were computed using *catRAPID omics* algorithm (Agostini *et al.*, 2013; Armaos *et al.*, 2021). This algorithm takes into account the calculation of secondary structure, hydrogen bonding and van der Waals contribution to predict protein-RNA interaction propensities with an excellent accuracy (Bellucci et al., 2011). It has been

developed to help the study on ribonucleoprotein complexes, since their experimental determination is a slow and challenging process. The algorithm can separate interacting vs non-interacting pairs with an area under the ROC curve of 0.78. The output is filtered according to the *Z*-score column, which is the interaction propensity normalized by the mean and standard deviation calculated over the reference RBP set (http://s.tartaglialab.com/static_files/shared/faqs.html#4). We selected three different thresholds in ascending order of stringency: *Z* greater or equal than 1.50, 1.75 and 2 respectively and for each threshold we then collected the proteins univocally bound to each fragment at each threshold.

*cat*RAPID performances were evaluated on the *Kamel W. et al. dataset.* We predicted the interactions of these proteins against the 30 SARS-CoV-2 different 1Kb regions and to have a unique score for each protein a normalized Z-score was calculated, that corresponds to the maximum-minimum Z-score among all the 30 fragments. *cat*RAPID *omics* calculations are available at http://crg-webservice.s3.amazonaws.com/submissions/2021-10/396382/output/index.html?unlock=75184cdc51. The performances were then calculated comparing the experimental fold-change and the normalized Z-score. At different cut-offs of the dataset the corresponding Area Under the Curve (AUC) of the corresponding Receiver Operating Characteristic (ROC) curve is finally computed.

Predictions of phase-separation

*cat*GRANULE (Bolognesi et al., 2016; Cid-Samper et al., 2018) was employed to predict the phase-separating propensity of proteins. Scores >0 indicate that a protein is prone to phase separate. Structural disorder, nucleic acid binding propensity and amino acid patterns such as arginine–glycine and phenylalanine–glycine are key features combined in this computational approach.

References

Agostini, F., Zanzoni, A., Klus, P., Marchese, D., Cirillo, D., and Tartaglia, G. G. (2013). catRAPID omics: a web server for large-scale prediction of protein-RNA interactions. *Bioinforma. Oxf. Engl.* 29, 2928–2930. doi:10.1093/bioinformatics/btt495.

Armaos, A., Colantoni, A., Proietti, G., Rupert, J., and Tartaglia, G. G. (2021). catRAPID omics v2.0: going deeper and wider in the prediction of protein-RNA interactions. *Nucleic Acids Res.*, gkab393. doi:10.1093/nar/gkab393.

Bellucci, M., Agostini, F., Masin, M., and Tartaglia, G. G. (2011). Predicting protein associations with long noncoding RNAs. *Nat. Methods* 8, 444–445. doi:10.1038/nmeth.1611.

Bolognesi, B., Lorenzo Gotor, N., Dhar, R., Cirillo, D., Baldrighi, M., Tartaglia, G. G., et al. (2016). A Concentration-Dependent Liquid Phase Separation Can Cause Toxicity upon Increased Protein Expression. *Cell Rep.* 16, 222–231. doi:10.1016/j.celrep.2016.05.076.

Cid-Samper, F., Gelabert-Baldrich, M., Lang, B., Lorenzo-Gotor, N., Ponti, R. D., Severijnen, L.-A. W. F. M., et al. (2018). An Integrative Study of Protein-RNA Condensates Identifies Scaffolding RNAs and Reveals Players in Fragile X-Associated Tremor/Ataxia Syndrome. *Cell Rep.* 25, 3422-3434.e7. doi:10.1016/j.celrep.2018.11.076.

Flynn, R. A., Belk, J. A., Qi, Y., Yasumoto, Y., Wei, J., Alfajaro, M. M., et al. (2021). Discovery and functional interrogation of SARS-CoV-2 RNA-host protein interactions. *Cell* 184, 2394-2411.e16. doi:10.1016/j.cell.2021.03.012.

Kamel, W., Noerenberg, M., Cerikan, B., Chen, H., Järvelin, A. I., Kammoun, M., et al. (2021). Global analysis of protein-RNA interactions in SARS-CoV-2-infected cells reveals key regulators of infection. *Mol. Cell* 81, 2851-2867.e7. doi:10.1016/j.molcel.2021.05.023.

Lee, S., Young-suk, Lee, Choi, Yeon, Son, Ahyeon, Park, Youngran, Lee, Kyung-Min, et al. (2021). The SARS-CoV-2 RNA interactome. *Mol. Cell* 81, 2838–2850. doi:10.1016/j.molcel.2021.04.022.

Schmidt, N., Lareau, C. A., Keshishian, H., Ganskih, S., Schneider, C., Hennig, T., et al. (2021). The SARS-CoV-2 RNA–protein interactome in infected human cells. *Nat. Microbiol.* 6, 339–353. doi:10.1038/s41564-020-00846-z.

The UniProt Consortium (2021). UniProt: the universal protein knowledgebase in 2021. *Nucleic Acids Res.* 49, D480–D489. doi:10.1093/nar/gkaa1100.

Vandelli, A., Monti, M., Milanetti, E., Ponti, R. D., and Tartaglia, G. G. (2020). Structural analysis of SARS-CoV-2 and predictions of the human interactome. *Nucleic Acids Res. Press*, 10.1101/2020.03.28.013789. doi:10.1101/2020.03.28.013789.

Supplementary Figures

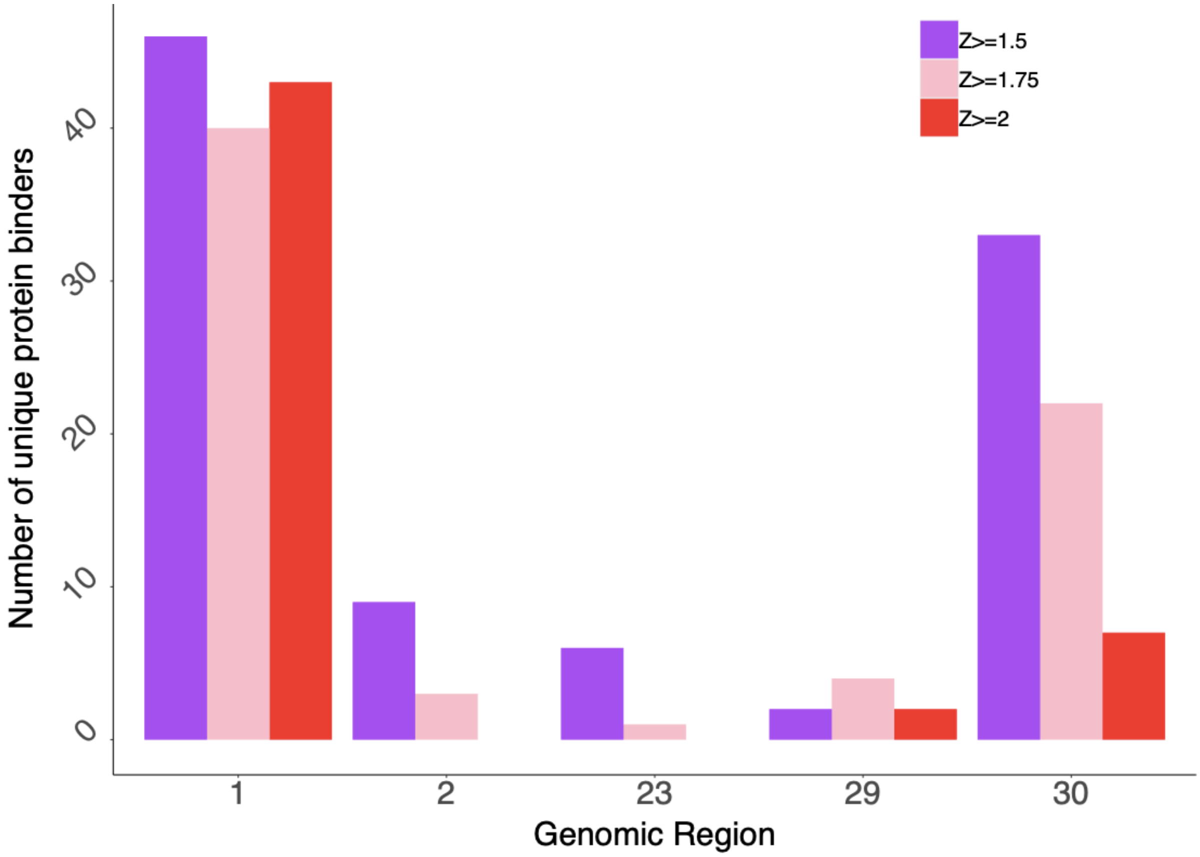

Supplementary Figure 1: Distribution of specific binders for Flynn R. et al. dataset (Flynn et al., 2021) at three different Z-score thresholds.

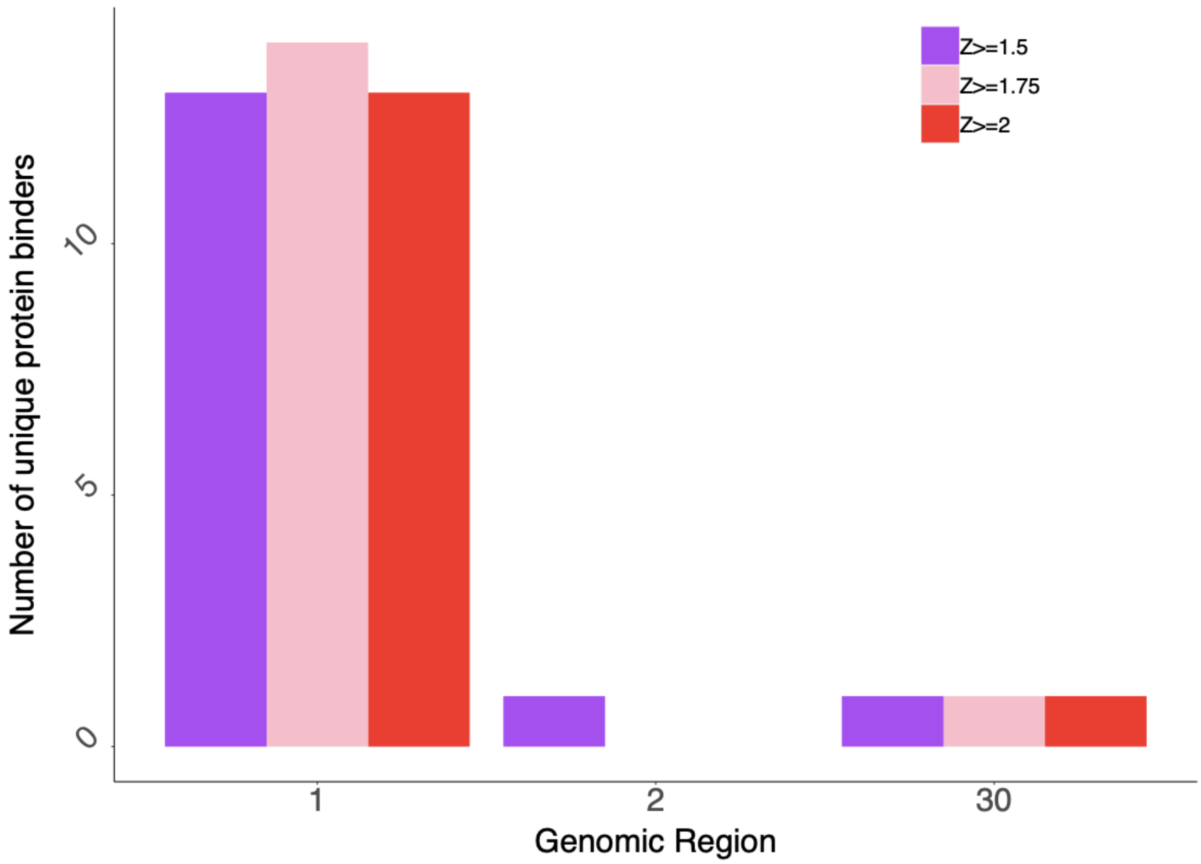

Supplementary Figure 2: Distribution of specific binders for Lee S. et al. dataset (Lee et al., 2021) at three different Z-score thresholds.

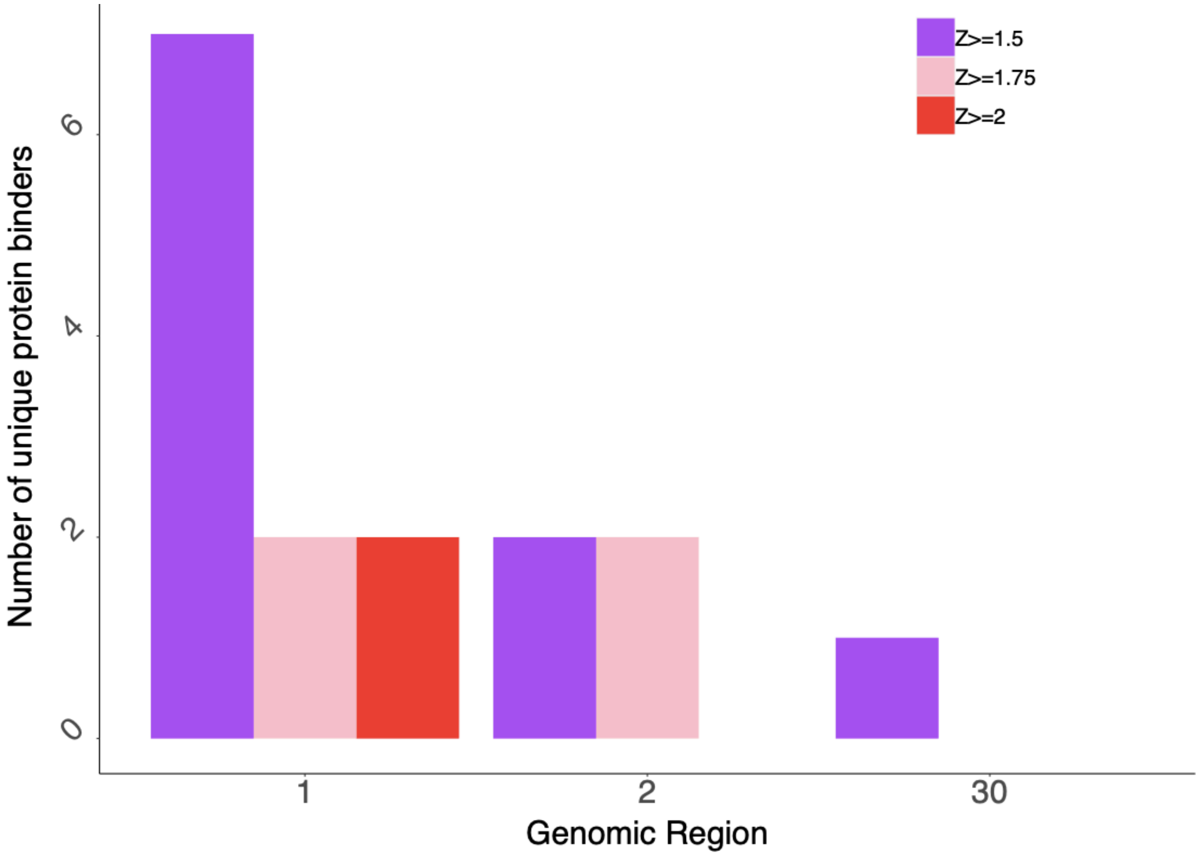

Supplementary Figure 3: Distribution of specific binders for Schmidt N. et al. dataset (Schmidt et al., 2021) at three different Z-score thresholds.
